# Outbreak of trichinellosis related to eating imported wild boar meat, Belgium, 2014

**DOI:** 10.2807/1560-7917.ES.2016.21.37.30341

**Published:** 2016-09-15

**Authors:** Peter Messiaen, Annemie Forier, Steven Vanderschueren, Caroline Theunissen, Jochen Nijs, Marjan Van Esbroeck, Emmanuel Bottieau, Koen De Schrijver, Inge C Gyssens, Reinoud Cartuyvels, Pierre Dorny, Jeroen van der Hilst, Daniel Blockmans

**Affiliations:** 1 Department of Infectious Diseases and Immunity, Jessa Hospital, Hasselt, Belgium; 2 BIOMED Research institute, Hasselt University, Hasselt, Belgium; 3 Department of Infectious Disease Control, Agency of Care and Health, Belgium; 4 Department of General Internal Medicine, University Hospital Leuven, Leuven, Belgium; 5 Department of Clinical Sciences, Institute of Tropical Medicine, Antwerp, Belgium; 6 Department of Gastro-enterology, St Trudo Hospital, St Truiden, Belgium; 7 Department of Infectious Disease Control, Agency of Care and Health, Belgium (affiliation when the work was performed); 8 Department of Epidemiology, University Antwerp, Antwerp, Belgium (current affiliation); 9 Radboud University Medical Center, Nijmegen, the Netherlands; 10 Department of Clinical Biology, Jessa Hospital, Hasselt, Belgium; 11 Department of Biomedical Sciences, Institute of Tropical Medicine, Antwerp, Belgium

**Keywords:** Trichinellosis, Outbreak management, Early warning system, Anti-parasitic treatment

## Abstract

Trichinellosis is a rare parasitic zoonosis caused by *Trichinella* following ingestion of raw or undercooked meat containing *Trichinella* larvae. In the past five years, there has been a sharp decrease in human trichinellosis incidence rates in the European Union due to better practices in rearing domestic animals and control measures in slaughterhouses. In November 2014, a large outbreak of trichinellosis occurred in Belgium, related to the consumption of imported wild boar meat. After a swift local public health response, 16 cases were identified and diagnosed with trichinellosis. Of the 16 cases, six were female. The diagnosis was confirmed by serology or the presence of larvae in the patients’ muscle biopsies by histology and/or PCR. The ensuing investigation traced the wild boar meat back to Spain. Several batches of imported wild boar meat were recalled but tested negative. The public health investigation allowed us to identify clustered undiagnosed cases. Early warning alerts and a coordinated response remain indispensable at a European level.

## Introduction

Trichinellosis is a parasitic zoonosis caused by nematodes of the genus *Trichinella.* The parasite infects domestic and wild animals and has a worldwide distribution [1]. The life cycle of the parasite consists of a domestic cycle in mainly pigs and a sylvatic cycle in a wider range of animals such as bears and wild boar [2-5]. Humans become infected after eating raw or undercooked meat from domestic pigs, horses or game containing *Trichinella* larvae [6-10]. The most important prevention measure is to freeze the meat or when preparing it, to ensure the core of the meat is cooked at a minimum of 67 °C, measured with a food thermometer, in order to kill the larvae. Different minimum temperatures and necessary duration of cooking are recommended according to the meat source [11].

*Trichinella* has unusual features, in comparison with other helminths. After ingestion, infective larvae are released from the muscular fibre and invade the epithelium of the host’s small intestine. Sexually mature adult worms produce larvae in the small intestine, which subsequently disseminate in the host and invade muscle tissue [12,13]. Once the parasite completes development in the muscle, it remains infective for months or years. The pathological mechanisms of disease are complex and are partly related to direct lesions caused by invasion of the parasite into the host’s muscle. A large inflammatory reaction mediated by eosinophils triggers numerous clinical manifestations during the acute stage of the disease [14].

The clinical picture is usually described by two stages: an intestinal stage within the first or second week after infection resulting in nausea or diarrhoea and a later muscular stage with periorbital oedema, myalgia or muscle weakness as the major symptoms [3]. The disease is mostly self-limiting: the adult worms live a mean of two to three weeks and the muscular phase is the end-stage of the infection [3]. However, major complications may arise during invasion of the muscle, including myocarditis, encephalitis and pulmonary superinfection. Fatalities have been described in infections with a high inoculum [15]. Cardiac involvement is the most frequent cause of death in human trichinellosis [16-18]. Although muscular symptoms usually subside within two to four weeks, even in mild infections, muscular fatigue may last up to six months. Treatment consists of administration of antiparasitic agents with or without systemic glucocorticoid treatment [3,9].

In Europe, four species of *Trichinella* (*T. spiralis*, *T. nativa*, *T. britovi* and *T. pseudospiralis*) are endemic in domestic and wild animals [19]. Since 1992, the European Union (EU) Council Directive 92/45 has required the examination of meat of wild boars (*Sus scrofa*), domestic pigs and horses for the presence of *Trichinella* species before processing and marketing [20,21]. Before implementation of the EU directives, high incidence rates of human trichinellosis were observed in eastern European countries (2.46–5.45 cases/100,000 persons/year), but they have decreased sharply in the past five years [22]. According to the European Centre for Disease Prevention and Control (ECDC), 320 confirmed human cases were reported in the EU during 2014 [23]. In Belgium, the last reported cases in humans after eating indigenous wild boar meat occurred in 1979 [24]. In Belgium, *Trichinella* infection has not been detected in domestic pigs or horses since 1992, although serological evidence has pointed towards the presence of *Trichinella* species in wild boar and foxes [25,26].

### The event

At the end of November 2014, 10 patients were admitted to three different hospitals in Belgium with fever, periorbital swelling, muscular pain and remarkable eosinophilia after eating wild boar meat in three different restaurants. A diagnosis of trichinellosis was confirmed by serology and PCR on the patients’ muscle biopsies, in which *T. spiralis* was identified. In order to determine the extent of the outbreak, to identify its source and to implement control measures, an epidemiological study was conducted. 

In Flanders, the northern region of Belgium, food-borne illnesses are notified to the Flemish Agency for Care and Health, which is responsible for investigating the source of disease and limiting its further spread. This is done in collaboration with the Federal Agency for the Safety of the Food Chain (FASFC), which operates nationwide to monitor and protect food safety. On 3 December, after the first diagnoses of human trichinellosis that day, both agencies were notified by email and other informal channels. Alerts were sent out regionally and all relevant public health authorities were informed. Through a ProMED Mail posting [27], this warning was communicated to the broader infectious disease community. The European Early warning and Response System (EWRS) and the Rapid Alert Safety for Food and Feed (RASFF) were alerted, to inform public health authorities in all EU Member States. Radio and television broadcasts and newspapers reported on the disease outbreak. Primary care physicians in the affected regions were asked to stay alert for patients with symptoms possibly related to trichinellosis and to ask symptomatic patients for details of potential exposure. Suspected cases had to be reported to the Flemish Agency for Care and Health.

## Methods

### Outbreak case definition

A probable case of trichinellosis was defined as a person who had consumed wild boar meat between 1 November and 6 December 2014 (two days after the first diagnosis and the start date of the outbreak investigation), with eosinophilia of > 500 cells/µL (norm: 0–450 cells/µL) with symptoms of myositis with or without fever (body temperature > 38 °C). Myositis was defined as muscle pain or muscle tenderness on physical examination and/or creatinine kinase levels > 200 international units (IU)/L (norm: 0–171 IU/L). 

A confirmed case was defined as a probable case with positive serology or seroconversion, detected by anti-*Trichinella* IgG, or the presence of intramuscular larvae in a muscle biopsy as demonstrated by histology and PCR.

### Laboratory analysis

The choice of diagnostic workup was at the discretion of the treating clinician and usually included blood counts, serum biochemical testing, electrocardiography, echocardiography, imaging studies and electromyography. Serological testing was performed at the National Reference Laboratory for Infectious and Tropical diseases at the Antwerp Institute of Tropical Medicine (ITM) using a commercially available assay based on excretory/secretory *Trichinella* antigens (Trichinella Microwell Serum ELISA, SciMedx Corporation, Denville, NJ, United States). ELISA-positive sera were confirmed by an in-house ELISA and western blot. Muscle biopsies were examined by the local pathologist. For this purpose, 3 μm sections were cut from formalin-fixed, paraffin-embedded muscle biopsy specimens and stained with haematoxylin and eosin. Portions of the biopsies were also sent to the National Reference Laboratory for Trichinella at ITM, for additional examination including trichinoscopy and magnetic stirrer artificial digestion [28]. After HCl-pepsin digestion, isolated larvae were characterised by multiplex PCR following DNA extraction from single larvae [29,30].

### Statistical analysis

Statistical analysis of the data was performed with SPSS 19. After normality testing using the Shapiro–Wilk test and assessment of the equality of variances with the Levene test, Student’s t-test was used to determine differences in continuous variables between subgroups. The differences between other epidemiological parameters were evaluated with Fisher’s exact test. An α-error of p < 0.05 was considered statistically significant.

### Trace-back investigation

The FASFC and the Flemish Agency for Care and Health conducted a trace-back investigation focusing on the supply chain of the suspected meat. A detailed questionnaire about time of consumption of wild boar meat, time and duration of symptoms and treatment modalities was sent to all confirmed cases. Clinical and laboratory data from the individual patient files were reviewed after obtaining informed consent. Serological testing for trichinellosis was performed for asymptomatic persons accompanying confirmed cases at the restaurants and reporting the same consumption.

## Results

### Epidemiological characteristics of the cases

During the last 2 weeks of November 2014 and the first 2 weeks of December 2014, 16 patients were identified as confirmed cases of trichinellosis. They all reported eating wild boar meat during the first week of November in three restaurants in the Belgian provinces of Limburg and Antwerp. The exact date of meat consumption was known for all the cases. Their median age was 37 years (interquartile range (IQR): 31–48; standard deviation (SD): 11); six were female (Table). Two subgroups were distinguished according to the type of exposure: cases who had eaten a full dish of slowly roasted wild boar fillet (classed as ‘severe’ exposure, n = 10) and those who had eaten small portions of slowly roasted wild boar fillet or wild boar stew (classed as ‘mild’ exposure, n = 6). 

**Table:** Characteristics of trichinellosis cases according to level of exposure^a^, Belgium, November–December 2014 (n = 16)

Characteristics	Number of cases^b^ among all cases n = 16	Number of cases^b^ among those with severe exposure^a^ n = 10	Number of cases^b^ among those with mild exposure^a^ n = 6	P value^c^
Median age in years (IQR)	37 (31–48)	47 (34–50)	30 (20–39)	0.02
Female	6	3	3	0.61
Median time to symptom onset after eating wild boar meat, in days (IQR)	13 (8–22)	9 (8–13)	22 (21–23)	< 0.00
Intestinal-stage gastrointestinal symptoms	6	4	2	1.00
Symptoms reported at presentation				
Fatigue	16	10	6	1.00
Fever	14	9	5	1.00
Night sweats	14	10	4	0.12
Periorbital oedema	14	9	5	1.00
Ophtalmological inflammation	14	9	5	1.00
Photophobia	6	4	2	1.00
Headache	12	7	5	1.00
Muscular pain	14	9	5	1.00
Abdominal pain	5	3	2	1.00
Rash	1	1	0	1.00
Lymphadenopathy	1	1	0	1.00
Outcome				
Hospitalisation	10	7	3	0.65
Myocarditis	4	4	0	0.23
Complete recovery	15	9	6	1.00

IQR: interquartile range.

^a^ Severe exposure was defined as having eaten a full dish of slowly roasted wild boar fillet. Mild exposure was defined as having eaten small portions of slowly roasted wild boar fillet or wild boar stew.

^b^ Unless otherwise specified.

^c^ Severe exposure vs mild exposure.

**Figure 1:**
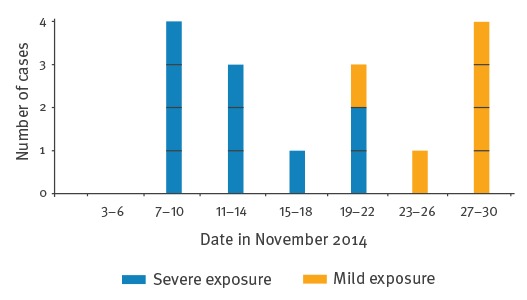
Epidemic curve of a trichinellosis outbreak, Belgium, November–December 2014 (n = 16)

**Figure 2:**
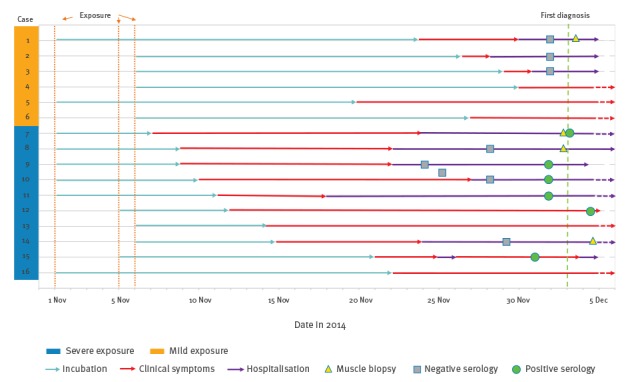
Timeline showing exposure, incubation period and diagnostic examinations, trichinellosis outbreak, Belgium, 1 November–6 December 2014 (n = 16)

None of the cases reported eating other game meat during the investigation period. The epidemic curve of the outbreak is shown in Figure 1 and a timeline with data on exposure, incubation period, and clinical and laboratory data are presented in Figure 2.

The number of new cases is displayed according to the date of symptom onset, per time frame of four days, according to the type of exposure. Severe exposure was defined as having eaten a full dish of slowly roasted wild boar fillet; mild exposure was defined as having eaten small portions of slowly roasted wild boar fillet or wild boar stew.

Cases 1–6 had mild exposure (defined as having eaten small portions of slowly roasted wild boar fillet or wild boar stew); Cases 7–16 had severe exposure (defined as having eaten a full dish of slowly roasted wild boar fillet).

Serological tests were performed after the initial diagnosis was made by muscle biopsy, in some cases retrospectively. Some cases were diagnosed or remained hospitalised after 5 December (shown as horizontal dotted lines).

### Clinical and laboratory data

The cases’ first symptoms appeared after a mean period of 15 days (range: 6–24; SD: 7) post-exposure. Cases with severe exposure had a significantly shorter incubation period compared with those with mild exposure: mean 10 days (range: 6–21; SD: 5) vs 22 days (range: 19–24; SD: 2), p < 0.000 (Table). The most frequent symptoms are summarised in the Table. Periorbital oedema lasted on average six days (range: 2–13 days; SD: 3). Muscular pain or tenderness was reported, ranging from moderate to severe and involved both upper and lower extremities (8/16) or was limited to the lower limbs (6/16). Symptoms related to the intestinal stage were reported by five cases. A total of 10 cases were hospitalised for a mean period of 14 days (range: 2–75; SD: 21); three required admission to an intensive-care unit.

Six cases initially presented with neurological complaints, including photophobia, neck stiffness and headache. Brain computed tomography (CT) of all cases showed no abnormalities. Three underwent lumbar puncture: their cerebrospinal fluid showed no cellular or biochemical abnormalities and bacterial cultures remained negative. One of the three who underwent lumbar puncture developed generalised muscle weakness 10 days after exposure, which remained severely debilitating two months later despite treatment with albendazole and corticosteroids. This case, a man in his early 50s, had, however, underlying conditions, which could obscure the clinical picture. A full-body positron emission tomography/CT and electromyography analysis were consistent with diffuse myositis [31]. The persisting symptoms included tremor, impaired coordination, fine motor control and loss of strength in the main muscle groups. 

Laboratory analysis of all cases showed marked eosinophilia, reaching a peak on four weeks after exposure, with a mean of 34% (range: 7–65%; SD: 13) white blood cells (norm: 1–6%). Serum creatinine kinase (CK) levels were elevated in all but two cases (mean: 666 units (U)/L (range: 101–1,564; SD: 450; norm: < 171 U/L), with a peak concentration coinciding with maximum eosinophilia levels.

In four of five cases tested for troponin T, elevated levels pointed to myocarditis. Electrocardiogram analysis pointed towards myocarditis in four cases. One such case had signs of anteroseptal myocardial oedema without a dynamic gradient on transthoracic ultrasound evaluation, which had normalised at a second examination three weeks later. Serial follow-up of troponin levels in three cases showed a prolonged elevation compared with CK levels.

All cases received anthelmintic treatment, consisting of oral mebendazole 300–500 mg every eight hours (n = 14) or albendazole 400mg every 12 hours (n = 2) for 14 days. The hospitalised cases (n = 10) received methylprednisolone for a minimum of two days. There was a mean of 32 days (range: 26–39; SD: 4) between exposure and treatment start. Clinical improvement was observed after 48–72 hours after treatment. All but the above-mentioned case had an uneventful recovery.

### Serology and microbiological identification

The clinical suspicion of *Trichinella* infection was confirmed by serology in all 16 cases. Biopsies of quadriceps muscle from three cases, weighing 0.24 g, 0.26 g and 0.30 g, revealed lymphohistiocytosis, eosinophilic infiltration and first-stage *Trichinella* larvae, which were identified as *T. spiralis* by multiplex PCR. One additional biopsy revealed eosinophilic myositis without detection of larvae. 

Three persons who ate slowly roasted wild boar fillet or wild boar stew at the same time in the same restaurants as eight of the cases stayed healthy without developing any symptoms. Paired samples tested serologically at five and nine weeks after consumption of the dish remained negative.

### Source tracing

The FASFC conducted an investigation, focusing on five suppliers of wild boar meat, in collaboration with the three restaurants involved. On inspection, there was evidence that the cold chain had been respected in all three restaurants. The investigation identified a single distributor of wild boar meat, imported from a certified supplier in north-eastern Spain. As there was a delay of several weeks between eating the suspected meat and the start of the investigation, no Spanish wild boar meat from this distributor remained in the restaurants where the cases had eaten. A week before the cases had eaten the suspected meat, all three restaurants received supplies of wild boar meat of the same batch on the same day. One supplier still had meat from the same batch. The remaining meat from this batch, as well as other batches from the same Spanish exporter, were recalled. A total of 58 samples from 21 different batches were examined by magnetic stirrer artificial digestion at the ITM, using 100 g per sample in 1g portions. None of these samples contained *Trichinella* larvae.

As a precaution, the FASFC decided that all sampled batches of Spanish wild boar meat had to undergo heat treatment at 84 °C for 640 min, using a protocol adapted to the final product and application of the meat. Remaining meat from the suspected batch was destroyed.

## Discussion

Despite the EU directive, which requires wild boars hunted for commercial purpose to be examined for *Trichinella larvae*, an infection risk for humans remains due to important reservoirs (wild carnivore mammals) [1,20,22]. The source of infection of this outbreak pointed towards wild boar meat imported from Spain, where *Trichinella* sp. infection is still endemic in the wild boar population [32,33]. According to ECDC epidemiological data, trichinellosis was most prevalent in eastern Europe (Romania, Bulgaria, Lithuania and Latvia) mainly due to eating domestic pork. In mediterranean Europe, Italy and Spain reported respectively 33 and 10 cases in 2012 [22]. Several outbreaks in these two southern European countries have been reported in the past 10 years [34-36].

No *Trichinella-*positive samples were found in the remaining imported wild boar meat. No remaining supplies of the suspected batch could be retrieved from the restaurants involved, due to the timeframe of the recall operation. The clinical pattern of the disease in humans often leads to a diagnosis several weeks after infection, which hampers source-tracing efforts. The authorities and controlling agencies in Spain reported no irregularities in the suspected slaughterhouse. No other *Trichinella* outbreaks were reported in the EU during the same time period. However, this cluster of infections highlights the importance of European-wide monitoring and an early warning system. Swift action by local networks and agencies is critical when a food-borne outbreak is detected. In this outbreak, the EWRS and RASFF made it possible to communicate efficiently and inform other potentially affected regions. Currently, the large EU-funded research Platform for European Preparedness Against (Re-) emerging Epidemics (PREPARE) is aiming to harmonise the response to severe infectious disease outbreaks and assemble real-time evidence for clinical management of patients. It should be noted, however, that due to the long incubation period and the delay of seroconversion in parasitic food-borne infections [3], mounting a rapid response to an outbreak is often difficult.

Although *Trichinella* larvae can be destroyed by heating [3], the preparation of boar meat remains problematic given the culinary habit of serving and eating meat that is not always fully cooked. In this outbreak, the cases who consumed a full dish of slowly roasted wild boar fillet seemed to have been exposed to a higher degree than those who ate wild boar stew, prepared at higher temperatures and for a longer duration. The recommendations of the United States Centers for Disease Control and Prevention (CDC) regarding prevention of trichinellosis stress the use of a food thermometer to evaluate the internal meat temperature [11].

It has been suggested that the severity of the clinical features of trichinellosis is proportional to the number of larvae ingested [14]. In this outbreak, cases with severe exposure developed symptoms significantly earlier and showed a trend towards more severe clinical presentation, with more gastrointestinal complaints and cardiological complications compared with cases with mild exposure.

The overall clinical picture was consistent with the typical pattern as reported in literature: signs and symptoms appeared one to four weeks after exposure and included almost always the classical triad of fever, periorbital oedema and muscular pain or tenderness [14]. Four of the 16 patients had symptoms of myocarditis, consistent with earlier studies investigating cardiac involvement in human trichinellosis [16-18]. There was evidence of ECG changes in 56% of cases in 154 cases in one study [37]. *Trichinella* larvae do not encyst in heart muscle cells: myocarditis is a consequence of the transient passage of larvae and the resulting eosinophilic infiltration in myocardial cells. In some of our cases, elevated troponin levels persisted after normalisation of CK, indicating a possible underestimation of myocarditis severity when only CK is measured. Our observation suggests that troponin measurement might be relevant as part of the initial assessment and follow-up of trichinellosis, together with conventional electrographic and ultrasound cardiac monitoring.

None of the cases had encephalitis, but some had symptoms suggestive of meningismus (photophobia, neck stiffness), although this was not confirmed as meningitis. In hindsight, the symptoms of photophobia, eye inflammation or visual disturbances could be related to ocular muscle involvement and larval invasion rather than neurological pathology.

Most patients (15/16) recovered rapidly after administration of antihelminthic treatment and/or corticoids. However, only a few randomised controlled trials and observational studies are available to guide treatment modalities. Several questions remain regarding the need for and choice or dosage of antihelminthics more than 4–6 weeks after exposure [3,38-40]. In contrast, early administration of antiparasitic treatment seems crucial to prevent or reduce trichinellosis symptoms. Recent evidence suggests that post-exposure prophylaxis within one week after infection should be strongly encouraged because the development of symptoms might be completely prevented [41].

Our report also shows the importance of obtaining a detailed food consumption history as quickly as possible when a cluster of infections with a similar clinical pattern is observed. With the combination of coordinated actions and communication, we identified a total of 16 cases, including seven who did not seek medical attention immediately. It is possible that additional cases with milder or no symptoms were missed. Although no active outreach to all potentially exposed persons was undertaken, we are confident that the large media coverage and intensive communication allowed the passive detection of most symptomatic patients. The requests for serological tests for trichinellosis at the ITM surged up to 10-fold in the immediate aftermath of the disease outbreak: none of those tests were positive.

## Conclusion

Several important lessons can be learnt from this outbreak, the largest reported to date in Belgium. Although a rare disease in western Europe, *Trichinella* remains a threat to food chain safety in 2016 despite measures taken at a European level. In countries where the prevalence of *Trichinella* in wildlife or domestic pork is still high, strict application of EU regulations and adequate control in slaughterhouses might be appropriate. In case of human infections, a transnational early warning system is important to alert the appropriate authorities, who can take swift action and control further spread of an outbreak.

Clinician’s awareness of the features suggestive of trichinellosis, particularly in clustered cases, can hasten early diagnosis, prevent complications and even lead to administration of effective post-exposure prophylaxis in other potentially exposed persons. The role of serial troponin measurements, in addition to CK levels, should be further explored as a marker of disease severity. 

Finally, there is a need for more research and clinical trials to establish sound treatment guidelines for trichinellosis.
